# Supplementary material for: Outcome of Irrigation and Debridement with Topical Antibiotic Delivery Using Antibiotic-Impregnated Calcium Hydroxyapatite for the Management of Periprosthetic Hip Joint Infection
Source: Antibiotics (Basel). 2023 May 21;12(5):938. doi: 10.3390/antibiotics12050938 (PMC10215927; doi:10.3390/antibiotics12050938)
Supplement: Supplementary file 1 [file antibiotics-12-00938-s001.zip › antibiotics-2361105-supplementary.pdf]

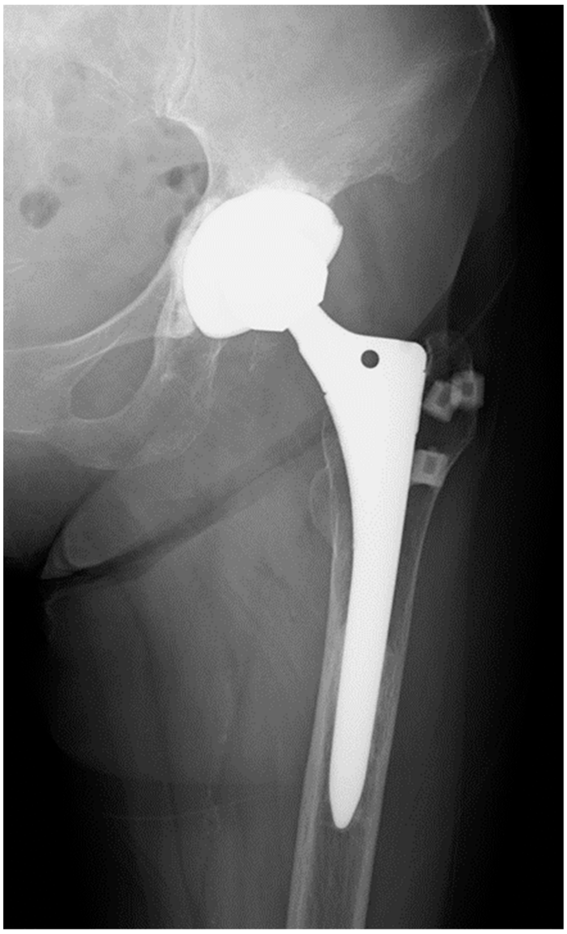

Figure S1. Radiographs of the left hip of a 74-year-old woman.

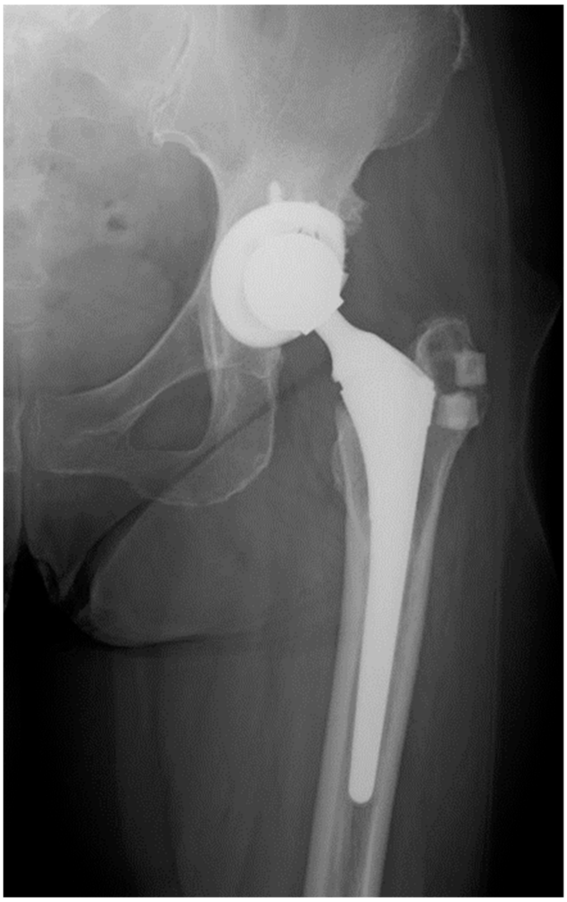

Figure S2. Radiographs of the left hip of a 63-year-old woman.

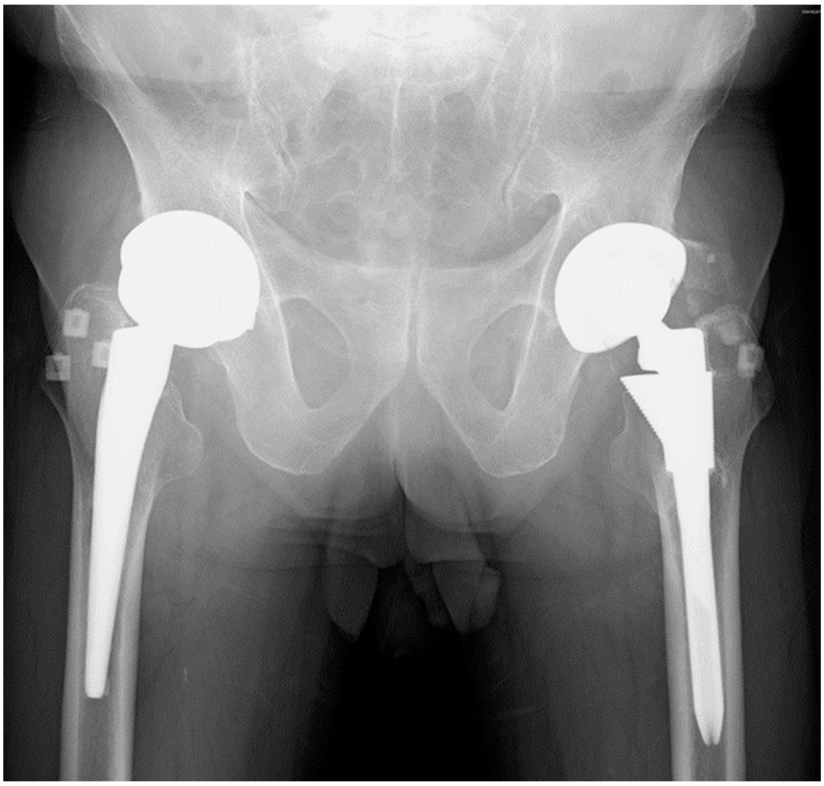

Figure S3. Radiographs of the both hips of a 71-year-old man.

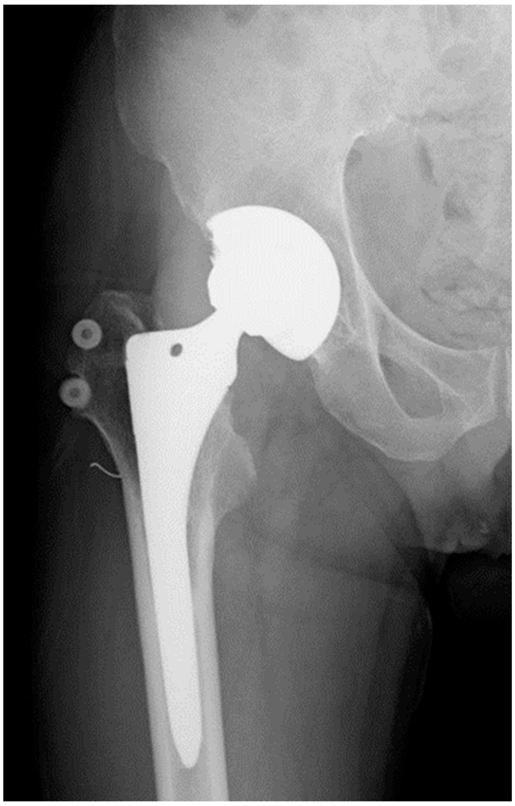

Figure S4. Radiographs of the right hip of a 57-year-old man.

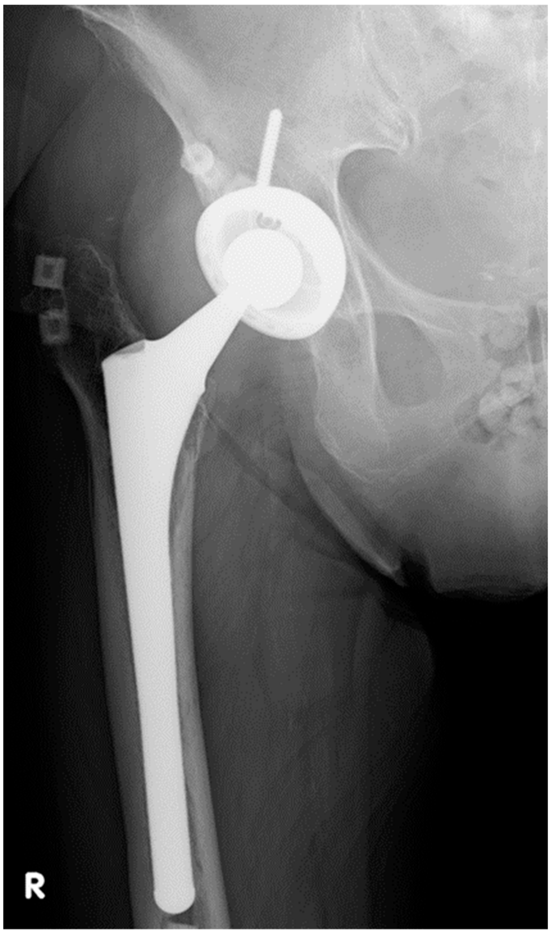

Figure S5. Radiographs of the right hip of a 69-year-old woman.
